# Minimal residual disease- and graft-vs.-host disease-guided multiple consolidation chemotherapy and donor lymphocyte infusion prevent second acute leukemia relapse after allotransplant

**DOI:** 10.1186/s13045-016-0319-5

**Published:** 2016-09-15

**Authors:** Chen-Hua Yan, Yu Wang, Jing-Zhi Wang, Yu-Hong Chen, Yao Chen, Feng-rong Wang, Yu-Qian Sun, Xiao-Dong Mo, Wei Han, Huan Chen, Xiao-hui Zhang, Lan-Ping Xu, Kai-Yan Liu, Xiao-Jun Huang

**Affiliations:** 1 Beijing Key Laboratory of Hematopoietic Stem Cell Transplantation, Peking University Institute of Hematology, Peking University People’s Hospital, Xi Zhimen South Street No. 11, Beijing, 100044 China; 2 Collaborative Innovation Center of Hematology, Xi Zhimen South Street No. 11, Beijing, 100044 China

**Keywords:** Allogeneic hematopoietic stem cell transplant, Leukemia relapse, Acute leukemia, Donor lymphocyte infusions, Minimal residual disease, Graft-vs.-host disease

## Abstract

**Background:**

Persons with acute leukemia relapsing after allotransplant and who respond to anti-leukemia interventions are at high risk of a second relapse. We studied the impact of minimal residual disease (MRD)- and graft-vs.-host disease (GvHD)-guided multiple consolidation chemotherapy and donor lymphocyte infusions (DLIs) to prevent second relapse in patients with acute leukemia relapsing post-transplant and who achieved complete remission after induction chemotherapy and DLI.

**Methods:**

Forty-seven subjects with acute leukemia relapsing after an allotransplant and who achieved complete remission after post-relapse induction chemotherapy and DLI were eligible. The use of consolidation chemotherapy and DLI was guided by the results of MRD testing and whether or not DLI caused acute and/or chronic GvHD. Outcomes were compared with those of 34 similar historical controls who did not receive consolidation chemotherapy and DLIs after induction chemotherapy and DLI.

**Results:**

One-year cumulative incidence of relapse (CIR; 22 % 95 % confidence interval (10, 35 %) vs. 56 % (39, 73 %); *P <* 0.0001), leukemia-free survival (LFS; 71 % (57, 84 %) vs. 35 % (19, 51 %); *P <* 0.0001), and survival (78 % (66, 90 %) vs. 44 % (27, 61 %); *P <* 0.0001) was significantly better in subjects than controls. In multivariate analyses, no chronic GvHD after therapy (hazard ratio (HR) = 3.56 (1.09, 11.58); *P =* 0.035) and a positive MRD test after therapy (HR = 21.04 (4.44, 94.87); *P <* 0.0001) were associated with an increased CIR.

**Conclusion:**

These data suggest MRD- and GvHD-guided multiple consolidation chemotherapy and DLIs reduce CIR and increase LFS and survival compared with controls in persons relapsing after allotransplant for acute leukemia.

**Trial registration:**

ChiCTR-ONC-12002912. Donor lymphocyte infusion for the treatment of leukemia relapse following allogeneic hematopoeitic stem cell transplant.

## Background

Leukemia relapse is still a major problem after allotransplants for acute leukemia [1, 2]. Therapy of post-transplant relapse includes stopping immune suppression and giving anti-leukemia chemotherapy and donor lymphocyte infusions (DLIs). Schmid et al. reported a 34 % complete remission rate from chemotherapy and DLI in 171 persons relapsing after an allotransplant for acute leukemia with a 2-year survival of 21 ± 3 % SD [3]. We recently reported a 64 % (95 % confidence interval (CI), 50–76 %) complete remission rate and 36 % (23, 49 %) 1-year leukemia-free survival (LFS) and 20 % (9, 33 %) 2-year LFS in 50 persons relapsing after an allotransplant [4]. Clearly, these results need improvement.

In persons receiving an allotransplant for acute leukemia, we reported an association between a positive minimal residual disease (MRD)-test after transplant and an increased risk of subsequent relapse [5–8]. Furthermore, in persons relapsing after an allotransplant and who achieve complete remission after induction chemotherapy and DLIs, the association between a positive MRD test and an increased risk of a second relapse was also reported in our previous study [4]. Also, persons developing chronic GvHD after receiving DLI for leukemia relapse after a first allotransplant have a lower likelihood of a second relapse compared with similar persons not developing chronic GvHD [4]. And, Mo et al. [9] also reported persons with chronic GvHD after DLI was associated with a greater frequency of a negative MRD test and lower likelihood of subsequent relapse compared with similar persons not developing chronic GvHD. Based on these data, we designed a study to determine whether giving additional consolidation chemotherapy and DLI might decrease likelihood of second relapse in persons without chronic GvHD or with a positive MRD test after initial post-relapse therapy with induction chemotherapy and DLI.

## Methods

### Eligibility

From January 1, 2013, to February 28, 2015, subjects receiving non-T cell-depleted an allotransplant at Peking University Institute of Hematology were eligible if they met the following criteria: (1) acute leukemia without t(9;22); (2) relapse after an allotransplant; (3) had full or partial-donor chimerism; and (4) received re-induction chemotherapy and DLI and achieved a complete remission. The study was approved by the Ethics Committee of Peking University People’s Hospital, and written informed consent was obtained from all subjects in accordance with the Declaration of Helsinki. The study is registered at http://www.chictr.org.cn as ChiCTR-ONC-12002912.

### Study-design and protocol

#### Subjects

From January 1, 2013, to February 28, 2015, 87 consecutive subjects ages 4–58 years relapsing after an allotransplant were screened for eligibility. Eight received only supportive care and nine received chemotherapy but not DLI because no donor was available (*N* = 6) or because there were no residual donor cells at time of relapse (*N* = 3). Forty-seven (67 % (56, 77 %)) of the remaining 70 subjects receiving re-induction chemotherapy and DLI to control relapse post-transplant achieved a complete remission and were eligible for this study. Controls were selected from among 69 similar subjects relapsing after allotransplant at our center from January 1, 2000, to December 31, 2008. We excluded five receiving only supportive care, seven who did not receive DLI because no donor was available (*N* = 4) or because there were no residual donor cells at time of relapse (*N* = 3), and three who received a second allotransplant. In the remaining 54 subjects receiving re-induction chemotherapy and DLI, 34 (63 % (50, 75 %)) subjects achieved a complete remission and served as controls.

#### Therapy-protocol

**Fig. 1:**
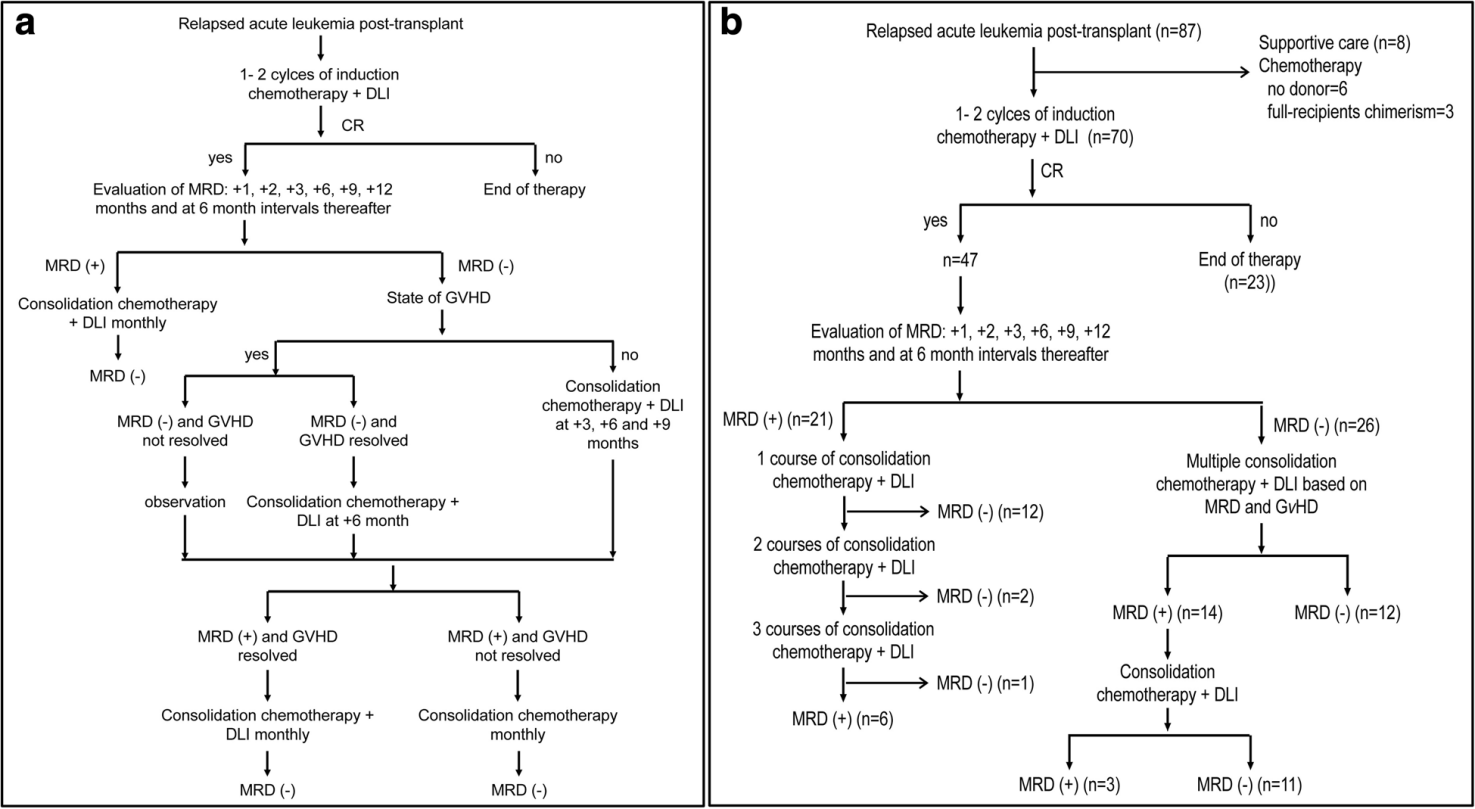
The diagram of intervention strategy. **a** The diagram of intervention strategy. **b** The diagram of patient subgroups

Post-transplant immune suppression was discontinued immediately upon relapse. All subjects then received induction chemotherapy (see below). DLI was given 48–72 h after completing chemotherapy (see below). Subjects not achieving a complete remission after a second course of induction chemotherapy and DLI were excluded. Subjects achieving a complete remission had MRD testing at 1, 2, 3, 6, 9, and 12 months and at 6-month intervals thereafter. Consolidation chemotherapy and additional DLIs (see below) were given based on the results of MRD testing and whether the subjects developed GvHD. Subjects with a positive MRD test received consolidation chemotherapy and DLIs monthly until the MRD test became negative. If white blood cells (WBC) counts recovered within 30 days, consolidation chemotherapy and DLIs were given monthly until MRD test became negative; if WBC counts recovered after 30 days, consolidation chemotherapy and DLIs were given when WBC counts recovered post-chemotherapy. Subjects with a negative MRD test and no GvHD received consolidation chemotherapy and DLIs at 3, 6, and 9 months. If MRD test was persistently negative and GvHD never resolved, consolidation chemotherapy and DLI was not given. If the MRD test was persistently negative but GvHD resolved, consolidation chemotherapy and DLI were given at 6 months. If the MRD test was positive again and GvHD resolved, consolidation chemotherapy and DLI were given monthly until the MRD test became negative again. If MRD test was positive again but GvHD never resolved, consolidation chemotherapy without DLI was given monthly until the MRD test became negative again. Interventions were continued until 1 year after achieving complete remission (Fig. 1a).

Induction chemotherapy in persons with acute myeloid leukemia (AML) was homoharringtonine, 2 mg/mE + 2/day for 5 days, aclacinomycin, 10 mg/mE + 2/day for 5 days and cytarabine, 100 mg/mE + 2/day for 5 days (HAA). Induction chemotherapy in persons with acute lymphoblastic leukemia (ALL) was cyclophosphamide, 800 mg/mE + 2/day for 2 days, vincristine 1 mg/mE + 2/day on day 1, daunorubicin, 40 mg/mE + 2/day for 3 days, and prednisone, 60 mg/day for 7 days (CODP). Subjects not achieving a complete remission after the first course of induction chemotherapy and DLI received a second course of induction chemotherapy with HAA or fludarabine, 30 mg/mE + 2/day for 5 days, cytarabine, 1 g every 12 h for 10 doses and granulocyte-colony stimulating factor (G-CSF), 300 μg/day for 6 days (FLAG) in subjects with AML and CODP or methotrexate (MTX), 1 g/mE + 2/day for 1 days and pegaspargase, 2000 U/mE + 2/day for 1 day in subjects with ALL. Consolidation chemotherapy in persons with AML was AA or HAA. Consolidation chemotherapy in persons with ALL was CODP or MTX, 1 g/mE + 2/day for 1 day.

DLIs used G-CSF mobilized blood cells followed by cyclosporine (CSA) or MTX to prevent GvHD. Details are reported [10, 11]. Median dose of mononuclear cells (MNC) for each infusion was 1.0 × 10E + 8/kg. Subjects could receive up to four courses of DLIs. Subjects receiving DLIs from a human leucocyte antigen (HLA)-haplotype-matched donor or an HLA-matched unrelated donor received CSA for 6 weeks after each infusion to prevent GvHD [10]. Subjects receiving DLIs from a HLA-identical related donor received CSA or MTX for 2–4 weeks after each infusion to prevent GvHD [11]. In subjects receiving DLI from a HLA-identical related donor with prior ≥grade 2 acute GvHD or ≥moderate chronic GvHD received CSA after DLI whereas others received MTX. The starting dose of CSA was 2.5 mg/kg/day and the dose was adjusted to maintain a plasma concentration 150–250 ng/ml. MTX, 10 mg, was given on days +1, +4, +8, +15, and +21.

#### MRD testing

We used to two strategies to test for MRD in bone marrow samples: (1) aberrant leukemia-associated immune phenotypes (LAIPs) detected by four-color flow cytometry (FCM) and (2) WT1 messenger RNA (mRNA) levels detected by polymerase chain reaction (PCR) [5–8, 12]. LAIPs were detected by four-color FCM. Different antibody combinations were used in B-ALL, T-ALL, and AML as described [5, 12]. 7.5 × 10E + 5 − 1 × 10E + 6 events were routinely analyzed. FCM-positive was defined as >0.01 % of cells with a LAIPs phenotype in ≥1 bone marrow samples. Sensitivity was 79 % and specificity was 85 % for persons with ALL [5]. WT1 mRNA level was evaluated by TaqMan-based RQ-PCR technology as described [6]. WT1-positive was defined as a transcript level >0.60 % in ≥1 bone marrow samples. Sensitivity was 69 % and specificity was 91 % for persons with AML [7]. Subjects with a positive LAIP or WT1 test were declared MRD test positive [7, 8].

#### Chimerism analysis

Chimerism analysis was performed by using DNA fingerprinting of short tandem repeats (STRs) from whole cell population in peripheral blood samples. If patients received transplant from sex-mismatched donors, chimerism analysis was also performed by using fluorescent in situ hybridization (FISH) for sex chromosomes in the bone marrow samples. Evaluations of chimerism were performed at the time of relapse post-transplant and at 1, 2, 3, 6, 9, and 12 months after induction chemotherapy and at 6-month intervals thereafter.

#### Transplants

Details of transplants including conditioning regimen, graft composition, GvHD prophylaxis, and supportive care are described [13–15]. Recipients of HLA-identical related transplants received cyclophosphamide, 1.8 g/mE + 2/day, for 2 days and 1 dose of 7.7 Gy total body radiation at 3.2 cGy/min or busulfan, 3.2 mg/kg/day IV for 3 days, and cyclophosphamide. Recipients of HLA-haplotype-matched related transplants and of HLA-matched unrelated transplants also received anti-human thymocyte immunoglobulin (ATG), 2.5 mg/Kg/d IV days −5 to −2 (Genzyme Corp, Boston, MA, USA). Grafts consisted of G-CSF mobilized bone marrow cells and peripheral blood cells. CSA, mycophenolate mofetil, and short-term MTX were given to prevent or modify GvHD.

### Definitions

Given that thrombocytic recovery could be postponed by factors other than leukemia and cytotoxic therapy (i.e., GvHD, virus, drugs), complete remission was defined as less than 5 % bone marrow blasts without evidence of dysplasia in bone marrow, no myeloblasts with Auer rods, no extra-medullary leukemia, and ANC ≥ 1 × 10E + 9/L. Leukemia relapse was defined as recurrence of ≥5 % bone marrow blasts or of ≥1 extra-medullary sites of leukemia. Neutrophil recovery was defined as an absolute neutrophil count (ANC) ≥ 0.5 × 10E + 9/L, and the time of neutrophil recovery was defined as the interval from the end of chemotherapy to the date of neutrophil recovery. Platelet recovery was defined as a platelet count ≥ 20 × 10E + 9/L for 7 consecutive days without transfusions, and the time of platelet recovery was defined as the interval from the end of chemotherapy to the date of platelet recovery. Survival was defined as interval from complete remission after post-relapse induction chemotherapy to death from any cause. LFS was defined as interval from the same start point to leukemia relapse or death whichever occurred first. Grading of acute GvHD and chronic GvHD used published criteria [16, 17].

### Statistics

The study was powered to detect 60 % LFS based on a reference rate of 35 % at 1 year derived using data from our center (unpublished). The primary end point was 1-year LFS. Secondary endpoints included incidence of acute and chronic GvHD, 1-year cumulative incidence of relapse (CIR), and survival.

CIRs, transplant-related mortality (TRM), and GvHD were calculated using a competing risk model. LFS and survival were calculated using the Kaplan-Meier method and compared using the log-rank test. Univariate analyses were performed using the *χ*^2^ test for categorical variables and the Mann-Whitney test for continuous variables. Multivariate analyses were performed using a Cox proportional hazards model. Potential interactions were tested, screened, and extracted from the analysis. Endpoint of follow-up for surviving subjects was February 28, 2016. Unless specified, all *P* values were two-sided and a *P* value <0.05 was considered significant. SPSS and R software packages were used for data analyses.

## Results

### Subject variables

Subject-related variables of trial subjects and controls are displayed in Table 1. Although most were similar, trial subjects were more likely to receive a HLA-haplotype-matched transplant (66 % (52, 78 %) vs. 56 % (39, 71 %); *P =* 0.037; Table 1). Besides, in 47 trial subjects achieving complete remission, platelet did not achieve 100 × 10E + 9/L at the time of complete remission in 7 subjects, and in 34 controls achieving complete remission, platelet did not achieve 100 × 10E + 9/L in 6 controls.

**Table 1:** Characteristics of patients in study group and historical group (*n* = 81)

Characteristics	Study group	Historical group	*P*
Patients’ number	47	34	
Age (years, range)	28 (4-58)	25 (7-57)	0.696
Gender			0.294
Male (%)	29 (62)	17 (50)	
Female (%)	18 (38)	17 (50)	
Diagnosis (%)			0.300
Acute myeloid leukemia	25 (53)	22 (65)	
Acute lymphoid leukemia	22 (47)	12 (35)	
Remission state pre-HSCT (%)			0.154
CR1	39 (83)	25 (73)	
CR2	5 (11)	9 (27)	
CR3	2 (4)	0 (0)	
NR	1 (2)	0 (0)	
Cytogenetic subgroups^a^ (%)			0.285
Intermediate	30 (64)	18 (53)	
Poor	10 (21)	6 (18)	
Not available	7 (15)	10 (29)	
Numbers of induction chemotherapies (%)			0.214
≤2	39 (85)	25 (74)	
>2	7 (15)	9 (26)	
Donor types (%)			0.037
HLA-identical related	11 (23)	15 (44)	
Haploidentical related	31 (66)	19 (56)	
Unrelated	5 (11)	0 (0)	
HLA-mismatch (%)			0.230
0 locus mismatch	4 (11)	0 (0)	
1 locus mismatch	3 (8)	4 (21)	
2 locus mismatch	10 (28)	7 (37)	
3 locus mismatch	19 (53)	8 (38)	
Donor-patient sex match (%)			0.377
Female-female	3 (6)	6 (18)	
Female-male	13 (28)	7 (21)	
Male-male	18 (38)	14 (41)	
Male-female	13 (28)	7 (21)	
ABO match (%)			0.994
Match	31 (66)	22 (65)	
Major mismatch	6 (13)	5 (15)	
Minor mismatch	6 (13)	4 (12)	
Major and minor mismatch	4 (9)	3 (9)	
Conditioning regimen (%)			0.635
TBI-based	3 (6)	1 (3)	
Bu-based	44 (94)	33 (97)	
Acute GvHD of grades 2–4 pre-DLI	14 (30)	9 (27)	0.744
Acute GvHD of grades 3–4 pre-DLI (%)	4 (9)	0 (0)	0.135
Chronic GvHD pre-DLI (%)	10 (21)	12 (35)	0.162
Moderate or severe chronic GvHD pre-DLI (%)	4 (9)	6 (18)	0.307
Interval from HSCT to relapse (day) (range)	204 (39-2180)	241 (40-2405)	0.737
BM blasts at the time of relapse (%) (range)	29 (7-93)	28 (9-92)	0.513
Chimerism at the time of relapse (%)			0.294
Full-donor chimerism	34 (72)	28 (82)	
Partial-donor chimerism	13 (28)	6 (18)	
Times of DLI after induction chemotherapy plus DLI			NA
1	21 (45)	0 (0)	
2	18 (38)	0 (0)	
3	7 (15)	0 (0)	
4	1 (2)	0 (0)	
MNCs in DLI (×10^8^/kg, range)	1.00 (1.00-1.50)	1.00 (0.70-1.70)	0.957
CD3^+^ cells in DLI (×10^8^/kg, range)	0.34 (0.15-0.64),	0.37 (0.15-0.74),	0.653
CD4^+^ cells in DLI (×10^8^/kg, range)	0.20 (0.10-0.42)	0.23 (0.09-0.31)	0.732
CD8^+^ cells in DLI (×10^8^/kg, range)	0.12 (0.06-0.28)	0.14 (0.05-0.31)	0.657
CD14^+^ cells in DLI (×10^8^/kg, range)	0.26 (0.04-0.48)	0.29 (0.09-0.61)	0.432
CD34^+^ cells in DLI (×10^6^/kg, range)	0.48 (0.14-1.44)	0.54 (0.12-1.58)	0.354

*HSCT* hematopoietic stem cell transplantation, *CR1* first complete remission, *CR2* second complete remission, *CR3* third complete remission, *NR* non-remission, *HLA* human leucocyte antigen, *TBI* total body irradiation, *Bu* busulfan, *GvHD* graft-vs.-host disease, *DLI* donor lymphocyte infusion, *BM* bone marrow, *NA* not available, *MNC* mononuclear cell

^a^The cytogenetic subgroups are according to the published data [35, 36]

Twenty-six trial subjects (55 % (41, 69 %)) were MRD test negative when they achieved a complete remission. Twelve others became MRD test negative after the first course of consolidation chemotherapy and DLI. Two more subjects became MRD test negative after the second course of consolidation chemotherapy and DLI, and one subject became MRD test negative after a third course of consolidation chemotherapy and DLI. Fourteen of the 26 subjects (54 % (35, 71 %)) with a negative MRD test when they achieved remission had ≥1 subsequent positive MRD tests. All received consolidation chemotherapy and DLIs, and 11 became MRD test negative. Finally, 38 trial subjects (81 % (67, 90 %)) were continuously MRD test negative (Fig 1b). Twenty-one trial subjects received one course of consolidation chemotherapy and DLI, 18 received two courses of consolidation chemotherapy and DLI, 7 received three courses of consolidation chemotherapy and DLI, and 1 received four courses of consolidation chemotherapy and DLI (Table 1). All trial subjects achieved neutrophil recovery with a median time of 17 days (range, 10–30 days). Forty-five trial subjects achieved platelet recovery with a median time of 20 days (range, 8–56 days). Besides, before disease relapse, 8 trial subjects received chemotherapy and DLI due to a positive MRD test.

### Relapse

**Fig. 2:**
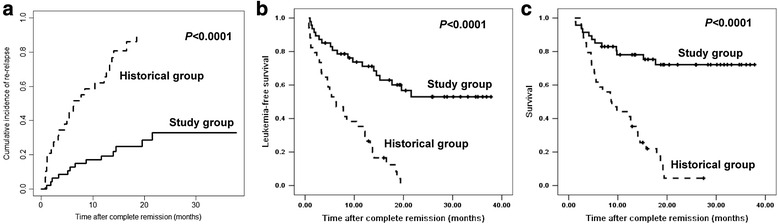
Comparison of outcomes after complete remission between study group and historical group. **a** Cumulative incidence of re-relapse after complete remission. **b** Leukemia-free survival (LFS) after complete remission. **c** Survival after complete remission. From January 1, 2013, to February 28, 2015, consecutive 47 patients received multiple consolidation chemotherapy and DLI and were finally included in study group. From January 1, 2000, to December 31, 2008, 34 patients only received induction chemotherapy plus DLI and were finally defined as historical group

Nine subjects had persistent MRD-positive tests including six after multiple courses of consolidation chemotherapy and DLI and three who were transiently MRD test negative. Seven relapsed and two other died of TRM. This contrasts with relapse in only 7 of 38 subjects who were consistently MRD test negative (*P =* 0.001). CIR was 22 % (10, 35 %) at 1 year and 35 % (19, 52 %) at 2 years (Table 2 and Fig. 2). In multivariate analyses, no chronic GvHD after DLI (HR *=* 3.56 (1.09, 11.58); *P =* 0.035) and persistent positive MRD tests after DLI (HR *=* 21.04 (4.66, 94.87); *P <* 0.0001; Table 3) were associated with increased relapse risks.

**Table 2:** Outcomes of patients in study group and historical group (*n* = 81)

Characteristics	Study group	Historical group	*P*
Patients’ number	47	34	
Cumulative incidence of relapse at 1 year (%)	22	56	<0.0001
Acute GvHD of grades 2–4 (%)	25	35	0.149
Acute GvHD of grade 3–4 (%)	11	15	0.366
Chronic GvHD (%)	52	33	0.039
Moderate or severe chronic GvHD (%)	49	23	0.005
TRM at 1 year (%)	9	6	0.064
LFS at 1 year (%)	71	35	<0.0001
Survival at 1 year (%)	78	44	<0.0001
Causes of mortality (*n*)	11	30	
Relapse (*n*)	7	25	
Infection (*n*)	3	3	
TMA (*n*)	1	0	
GvHD (*n*)	0	2	

*GvHD* graft-vs.-host disease, *TRM* treatment-related mortality, *LFS* leukemia-free survival, *TMA* thrombotic microangiopathy

**Table 3:** Univariate and multivariate analyses for re-relapse after complete remission

Characteristics	Relapse	
Univariate analysis	*P*	
Age	0.468	
Gender	0.521	
Diagnosis	0.211	
Remission status pre-HSCT	0.748	
Cytogenetic subgroups^a^	0.294	
Numbers of induction chemotherapies	0.654	
Donor types	0.735	
HLA-mismatch	0.090	
Donor-patient sex match	0.932	
ABO match	0.154	
Conditioning regimen	1.000	
Acute GvHD of grades 2–4 pre-DLI	0.726	
Chronic GvHD pre-DLI	0.703	
Interval from HSCT to relapse	0.695	
BM blasts at the time of relapse	0.443	
Chimerism at the time of relapse	0.467	
MNCs in DLI	0.388	
CD3^+^ cells in DLI	0.252	
CD4^+^ cells in DLI	0.242	
CD8^+^ cells in DLI	0.348	
CD14^+^ cells in DLI	0.209	
CD34^+^ cells in DLI	0.817	
Acute GvHD of grades 2–4 post-DLI	0.413	
Chronic GvHD post-DLI	0.002	
Persistent MRD-positive state post-DLI	0.001	
Multivariate analysis	*P*	*HR*
Persistent MRD-positive state post-DLI	<0.0001	21.04
No chronic GvHD post-DLI	0.035	3.56

*HSCT* hematopoietic stem cell transplantation, *HLA* human leucocyte antigen, *GvHD* graft-vs.-host disease, *DLI* donor lymphocyte infusion, *BM* bone marrow, *MNC* mononuclear cell, *MRD* minimal residual disease

^a^The cytogenetic subgroups are according to the published data [35, 36]

### GvHD

Nine trial subjects developed acute GvHD after DLI. Of the nine subjects, one developed grade 1 acute GvHD, five developed grade 2 acute GvHD, two developed grade 3 acute GvHD, and one developed grade 4 acute GvHD. Skin affected occurred in eight subjects, liver affected occurred in two, and intestinal tract affected occurred in four. Cumulative incidences of ≥grade 2 acute GvHD and ≥grade 3 acute GvHD were 25 % (15, 39 %) and 11 % (3, 22 %) (Table 2). Besides, 37 subjects developed chronic GvHD, 31 subjects developed ≥moderate chronic GvHD. Of the 37 subjects, 5 had a history of acute GvHD after DLI. Cumulative incidence of chronic GvHD and ≥moderate chronic GvHD at 1 year were 52 % (39, 65 %) and 49 % (34, 64 %) (Table 2). Cumulative incidences of chronic and ≥moderate chronic GvHD increased gradually with increased numbers of courses of consolidation chemotherapy and DLI: chronic GvHD: 1 course 35 % (18, 52 %) vs. 2 courses 72 % (57, 86 %) vs. 3–4 courses 100 % (83, 100 %); *P =* 0.002) and ≥moderate chronic GvHD: 1 course 30 % (19, 41 %) vs. 2 courses 70 % (55, 85 %) vs. 3–4 courses 100 % (92, 100 %); *P =* 0.003). In contrast, cumulative incidences of ≥grade 2 acute GvHD and ≥grade 3 acute GvHD were not significantly associated with numbers of courses of consolidation chemotherapy and DLI (*P =* 0.17 and *P =* 0.77).

### LFS and survival

Eleven trial subjects died, seven died of relapse, three died of infection, and one died of thrombotic microangiopathy. Median LFS was 23 months (range, 4–38 months). LFSs at 1 and 2 years were 71 % (59, 85 %) and 53 % (37, 69 %). Median survival was 32 months (range, 8–38 months). Survivals at 1 and 2 years were 78 % (66, 90 %) and 72 % (59, 86 %; Table 2 and Fig. 2).

### Comparison of study subjects and controls

Study subjects had a higher rate of persistent MRD-negative tests than controls (81 % (67, 90 %) vs. 9 % (3, 23 %); *P <* 0.0001). CIR in the study cohort was less than in controls (22 % (10, 35 %) vs. 56 % (39, 73 %); *P <* 0.0001). Cumulative incidences of ≥grade 2 and ≥grade 3 acute GvHD were similar to controls (*P =* 0.149 and *P =* 0.366). One-year cumulative incidences of chronic GvHD (52 % (39, 65 %) vs. 33 % (12, 54 %); *P =* 0.039) and ≥moderate chronic GvHD (49 % (34, 64 %) vs. 23 % (8, 38 %); *P =* 0.005) was significantly higher in subjects vs. control. One-year LFS was significantly better in subjects vs. controls (71 % (57, 84 %) vs. 35 % (19, 51 %), *P <* 0.0001). One-year survival was also better (78 % (66, 90 %) vs. 44 % (27, 61 %); *P <* 0.0001; Table 2 and Fig. 2).

## Discussion

We found MRD test results and GvHD-guided multiple consolidation chemotherapy and DLIs reduced CIR and improved LFS and survival compared with historical controls in patients with relapsed acute leukemia after allo-hematopoietic stem cell transplantation (HSCT). This was probably due to that the use of multiple consolidation chemotherapy and DLIs after induction chemotherapy and DLI could make more patients achieve and maintain a persistent negative MRD test. Although induction chemotherapy and DLI could make patients achieve complete remission in patients with relapsed acute leukemia after transplant, only 55 % of patients achieved a negative MRD test, and 54 % of these patients had subsequent positive MRD tests. But, after multiple consolidation chemotherapy and DLIs, 81 % of patients finally maintained a negative MRD test, compared with a 9 % of negative MRD test rate in historical control (*P <* 0.0001). Many studies already report a positive MRD test is associated with an increased relapse risk post-transplant [5–7, 12, 18, 19]. As well, based on the results of MRD tests, preemptive use of DLI could make patients achieve a negative MRD test and prevent relapse post-transplant in patients with standard risk acute leukemia [8]. Besides, multiple consolidation chemotherapy and DLIs also induced chronic GvHD and stronger graft-vs.-leukemia (GvL) effects. Our present study suggested that the incidence of chronic GvHD in study group was significantly higher than that in historical control (52 vs. 33 %, *P =* 0.039), as well as, the incidence of chronic GvHD post-DLI increased gradually with increased numbers of courses of consolidation chemotherapy and DLI (*P =* 0.002). Many researchers have already demonstrated that the development of chronic GvHD post-DLI was a favorable factor of CIR and survival in patients with relapsed acute leukemia after allo-HSCT [3, 4, 20, 21]. Therefore, all of these data suggested that the application of multiple consolidation chemotherapy and DLIs after induction chemotherapy and DLI could prevent the second relapse in patients with relapsed acute leukemia post-transplant, by inducing chronic GvHD and maintaining the negative MRD test.

Severe GvHD is a major risk of multiple consolidation chemotherapy and DLIs, which is usually correlated with higher TRM. However, we found that the incidences of ≥grade 2 acute GvHD and ≥grade 3 acute GvHD in study group were all similar to that in historical control (*P =* 0.149 and *P =* 0.366). And ultimately, there was no significant difference in the incidence of TRM between two groups (8.8 vs. 6.4 %, *P =* 0.064). A probable reason is due to the application of immunosuppressive agents for 2–4 weeks in patients receiving DLI from HLA-identical related donors and for 6 weeks in patients receiving DLI from haploidentical related donors or unrelated donors. Our previous study suggested that the duration of immunosuppressive agents used after DLI was the only risk factor for the development of ≥grade 3 acute GvHD after DLI (*P =* 0.018) and the cumulative incidence of ≥grade-3 acute GvHD in patients receiving immunosuppressive agents for ≥6 weeks was only 9.3 % [22]. Besides, the application of immunosuppressive agents for ≥6 weeks after DLI from haploidentical related donors and 2–4 weeks after DLI from HLA-identical related donors could preserve GvL effects at the same time could reduce the incidence of ≥grade 3 acute GvHD after DLI [10, 11]. As well, our present study also suggested that cumulative incidence of ≥grade 2 acute GvHD and ≥grade 3 acute GvHD were not significantly associated with numbers of courses of consolidation chemotherapy and DLI (*P =* 0.17 and *P =* 0.77), although cumulative incidences of chronic GvHD and ≥moderate chronic GvHD increased gradually with increased numbers of courses of consolidation chemotherapy and DLI (*P =* 0.003). Another probable reason is due to the use of G-CSF mobilized peripheral blood cells instead of unstimulated lymphocytes. Huang et al. [23, 24] reported that the application of G-CSF may modulate the polarization of T cells from a Th1 to a Th2 phenotype and indirectly induce T-cell hypo-responsiveness through the selective increase of DC2 cells and monocytes and the down-regulation of the CD28/B7 co-stimulatory signal. Moreover, Morris et al. [25] confirmed that using G-CSF during blood cell mobilization augments NK-T-cell-dependent CD8^+^ cytotoxicity and purportedly separates GvHD and GvL. Our previous study also suggested that compared with chemotherapy and DLI with unstimulated lymphocytes, chemotherapy and G-CSF mobilized peripheral blood cells infusion tended to be associated with a higher complete remission rate (7/9 vs. 3/5) and lower incidence of ≥grade 3 acute GvHD (0/9 vs. 1/5) in patients with relapsed hematological malignancies after HLA-identical related HSCT [26]. Therefore, although trial subjects received multiple consolidation chemotherapy and DLIs, no subjects died of GvHD. Another risk of multiple consolidation chemotherapy and DLIs is pancytopenia, which usually leads to higher infection-related mortality. Raiola AM et al. [21] found that chemotherapy and DLI with unstimulated lymphocytes was typically associated with a higher incidence of pancytopenia (18/100) and a longer duration of pancytopenia (median duration, 90 days). However, chemotherapy and G-CSF mobilized peripheral blood cells infusion was associated with a lower incidence of pancytopenia (2/57) [27]. In this study, all trial subjects achieved neutrophil recovery with a median time of 17 day, 45 trial subjects achieved platelet recovery with a median time of 20 days, and only 3 subjects died of infection. All the results confirmed the safety of chemotherapy and DLI which used G-CSF mobilized peripheral blood cells followed by CSA or MTX to prevent GvHD. As well, due to the improvement of diagnosis and treatment of GvHD and infectious disease post-transplant, it also ensured the successful use of multiple consolidation chemotherapy and DLIs. Moreover, because multiple consolidation chemotherapy and DLIs were given based on the results of MRD test and GvHD post-DLI, therefore, it avoided unnecessary TRM while could prevent second relapse in patients with relapsed acute leukemia post-transplant.

Besides, although multiple consolidation chemotherapy and DLIs were given, nine patients were still in a positive MRD test and seven out of nine patients relapsed again post-DLI. Mo et al. [9] also found that early-onset positive MRD test after DLI and persistent positive MRD test after DLI were usually associated with increased relapse risk (*P =* 0.001) and impaired LFS (*P =* 0.004). Therefore, for those patients with a positive MRD test post-DLI, more intensive therapy should be used so as to make them maintain a persistent negative MRD test. Mo et al. [28] found that in patients who were in a positive MRD test post-transplant, compared with DLI, interferon-α (IFN-α) could provide comparable rate of negative MRD test, relapse rate (27.3 % vs. 35.6 %, *P =* 0.514) and disease-free survival (68.2 vs. 60.0 %, *P =* 0.517). Furthermore, even in patients who had unsatisfied response to DLI, IFN-α could also provide a decreased level of MRD or a negative MRD test [29]. Besides, some articles have already suggested that second transplant could offered a chance of stable remission for patients with acute leukemia relapsing post-transplant and provided a 25–30 % of survival [30, 31]. Moreover, our previous study found that in 24 patients receiving second transplant to treat relapsed acute leukemia post-transplant, although 12 patients had no response to chemotherapy and DLI, second transplant still provided 55.7 % of CIR, 35.1 % of non-relapse mortality and 30.9 % of survival [32]. Therefore, for patients who had a persistent positive MRD test after two courses of consolidation chemotherapy and DLIs, DLI followed by IFN-α or second transplant were probably alternative methods. This needed to be investigated in future.

In addition, in this study, the complete remission rate (67 %, 47/60) is higher than that in article published before [3]. This is maybe due to the application of chemotherapy before DLI. Collins RH et al. [33] reported that complete remission rate in AML (*n* = 39) and ALL (*n* = 11) patients who had not received chemotherapy before DLI were only 15.4 and 18.2 %, respectively. But, in the article from Schmid C et al. [3], 75 % AML patients received chemotherapy before DLI and 35 % patients achieved complete remission. Besides, our previous study also suggested that chemotherapy and DLI provided a 64 % of complete remission rate in subjects with relapsed acute leukemia post-transplant [4]. Another reason is maybe due to the infusion of G-CSF mobilized peripherial blood cells infusion instead of unstimulate donor lymphocytes. Morris et al. [25] confirmed that using G-CSF during blood cell mobilization augments NK-T-cell-dependent CD8^+^ cytotoxicity. As well, our previous study suggested that compared with chemotherapy and DLI with unstimulated lymphocytes, chemotherapy and G-CSF mobilized peripherial blood cells infusion tended to be associated with a higher complete remission rate (7/9 vs. 3/5) in patients with relapsed acute leukemia after allo-HSCT [26]. In addition, Levine JE et al. [34] also reported that chemotherapy and DLI with G-CSF mobilized peripherial blood cells infusion could make 27 patients (27/57, 47 %) achieve complete remission.

Of course, there are some limitations to interpreting data from our study. The most important one is that historical cases were used as control, rather than randomized control. But, most variable were comparable between study group and historical control except for donor types (*P =* 0.037). In study group, more subjects receive a HLA-haplotype-matched transplant compared with historical control (66 % vs. 56 %, *P =* 0.037). However, the impact of this imbalance should be to increase rather than decrease the difficulties in interventions. As well, because multiple consolidation chemotherapy and DLIs were given based on the results of MRD tests and GvHD, therefore, it is difficult to perform randomized control study.

## Conclusion

These data confirmed that after induction chemotherapy and DLI, MRD test results and GvHD-guided multiple consolidation chemotherapy and DLIs reduced CIR and improved LFS and survival in patients with relapsed acute leukemia post-transplant.
