# Correction to: Minimal residual disease- and graft-vs.-host disease-guided multiple consolidation chemotherapy and donor lymphocyte infusion prevent second acute leukemia relapse after allotransplant

**DOI:** 10.1186/s13045-019-0715-8

**Published:** 2019-03-05

**Authors:** Chen-Hua Yan, Yu Wang, Jing-Zhi Wang, Yu-Hong Chen, Yao Chen, Feng-rong Wang, Yu-Qian Sun, Xiao-Dong Mo, Wei Han, Huan Chen, Xiao-hui Zhang, Lan-Ping Xu, Kai-Yan Liu, Xiao-Jun Huang

**Affiliations:** 1 Beijing Key Laboratory of Hematopoietic Stem Cell Transplantation, Peking University Institute of Hematology, Peking University People’s Hospital, Xi Zhimen South Street No. 11, Beijing, 100044 China; 2 Collaborative Innovation Center of Hematology, Xi Zhimen South Street No. 11, Beijing, 100044 China

**Correction to: J Hematol Oncol (2016) 9:87**

**https://doi.org/10.1186/s13045-016-0319-5**

The original article [1] contains two errors in the MRD Testing sub-section of the Methods:

1. The description, “…by four-color flow cytometry (FCM)” was incorrect. The correct description should be “…by eight-color flow cytometry (FCM)”.
2. The description, “LAIPs were detected by four-color FCM” was incorrect. The correct description should be “LAIPs were detected by eight-color FCM”.
